# Inflated Rubber Cylinder for Circular Suture of the Intestine

**Published:** 1898-05

**Authors:** 


### INFLATED RUBBER CYLINDER FOR CIRCULAR SUTURE OF THE INTESTINE.

Dr. W. S. Halsted, in the *Philadelphia Medical Journal*, Vol. 1, p. 63, finds that the disadvantages of methods of intestinal suture without mechanical aids have been disposed of by the employment of rubber cylinders. The operation is fully figured. The advantages of this cylinder in circular suture of the intestine are given as follows:

1. The vermicular action of the bowel is arrested over the bag, and the stitches can, consequently, be placed at regular and proper intervals.
2. The distended bag unrolls and spreads out to a fine edge the everted raw edge of the intestine, and enables the operator to place the stitches with great precision at the desired distance from this edge.
3. If a distended intestine is to be sutured to collapsed intestine (in strangulated hernia, ileus, etc.), or intestine of larger to intestine of smaller lumen (jejunum to ileum, duodenum to esophageal end of the stomach, etc.), the smaller may easily be expanded to fit the larger piece. This is perhaps the most important function of the cylinder.
4. The cylinder takes the place of at least two assistants.
5. It prevents escape of intestinal contents, and hence dispenses with the injurious clamps or the fingers of assistants.
6. The entire operation, exclusive of suture of the abdominal wall, can be performed on dogs in five or six minutes, and probably in less time.
7. Very little handling of the intestine itself by the operator is necessary. The tube from bag to syringe is used as a handle to rotate and elevate the parts to be united.
8. The operation could readily be performed without a single assistant.

